# Exploring survival-associated transcriptomic subtypes in ovarian cancer using RNAseq from FFPE tissues in a clinical trial cohort

**DOI:** 10.1016/j.tranon.2026.102740

**Published:** 2026-03-19

**Authors:** Maj K Kjeldsen, Frederik Otzen Bagger, Henrik Roed, Gitte-Bettina Nyvang, Charlotte Aaquist Haslund, Anja Oer Knudsen, Anne Krejbjerg Motavaf, Susanne Malander, Maarit Anttila, Gabriel Lindahl, Johanna Mäenpää, Maria Dimoula, Theresa Werner, Trine Zeeberg Iversen, Sakari Hietanen, Lars Fokdal, Hanna Dahlstrand, Line Bjørge, Michael Birrer, Mansoor Raza Mirza, Maria Rossing

**Affiliations:** a Department of Oncology, Copenhagen University Hospital, Rigshospitalet, Copenhagen, Denmark; b Center for Genomic Medicine, Copenhagen University Hospital, Rigshospitalet, Copenhagen, Denmark; c Nordic Society of Gynecological Oncology-Clinical Trial Unit, NSGO-CTU; d Department of Oncology, Odense University Hospital, Odense, Denmark; e Department of Oncology, Aalborg University Hospital, Aalborg; f Department of Oncology, Department of Clinical Science, Skåne University Hospital, Lund University, Lund, Sweden; g Department of Obstetrics and Gynecology, Kuopio University Hospital, Finland; h Department of Oncology, Linköping University Hospital, Linköping, Sweden; i Department of Biomedical and Clinical Sciences, Linköping University, Linköping, Sweden; j Tampere University and Cancer Center, Tampere University Hospital, Tampere, Finland; k Department of Oncology, Uppsala University Hospital, Uppsala, Sweden; l Huntsman Cancer Institute, University of Utah, Salt Lake City, UT, USA; m Department of Oncology, Herlev-Gentofte University Hospital, Herlev, Denmark; n Department of Obst & Gyn, Turku University Hospital and FICAN west, Turku Finland; o Department of Oncology, Vejle Hospital, University Hospital of Southern Denmark, Denmark; p Department of Oncology-Pathology, Karolinska Institutet and Theme Cancer, Karolinska University Hospital, Stockholm, Sweden; q Department of Obstetrics and Gynecology, Haukeland University Hospital, Bergen, Norway; r Centre for Cancer Biomarkers CCBIO, Department of Clinical Science, University of Bergen, Bergen, Norway; s Winthrop P. Rockefeller Cancer Institute, Little Rock, AR, USA; t Department of Clinical Medicine, Faculty of Health and Medical Sciences, University of Copenhagen, Copenhagen, Denmark

**Keywords:** Epithelial Ovarian Cancer (EOC), RNA sequencing (RNAseq), Overall Survival (OS), Transcriptomic subtypes, Differentially Expressed Genes (DEGs)

## Abstract

**Objective:**

Transcriptomic subtyping is not yet standardized for prognostic use in epithelial ovarian cancer (EOC). This study aims to validate RNA sequencing (RNAseq) from formalin-fixed, paraffin-embedded (FFPE) tissues and to evaluate survival-associated transcriptomic subtypes and differentially expressed genes (DEGs) in a clinical trial cohort.

**Methods:**

An exploratory post hoc analysis was conducted on FFPE samples from patients enrolled in the ENGOT-ov24/NSGO-AVANOVA1&2 trial. RNA was extracted and sequenced, and gene expression analysis was performed to classify subtypes using established, microarray-based, algorithms. Differentially expressed genes (DEGs) were identified based on survival groups, and survival outcomes were analyzed using Kaplan-Meier curves.

**Results:**

Of 96 eligible samples, 82 were included in the final analysis. Subtype classifications showed moderate agreement across RNAseq data formats. However, gene expression variability showed inconsistent concordance with clinical metadata and molecular subtypes. Eighteen genes were differentially expressed between long- and short-term survivors. Notably, DPEP3 and SLC14A1, were significantly upregulated in long-term survivors. Despite distinct expression patterns, no significant survival differences were observed between subtypes.

**Conclusions:**

This study demonstrates the feasibility of using RNAseq on FFPE tissue in EOC, while also highlighting challenges of applying microarray-based transcriptomic subtypes to RNAseq data. Transcriptomic analysis identified potential prognostic gene candidates but also highlighted the need to refine classification tools. Further research is essential to improve the molecular classification of EOC, thereby enhancing prognostic accuracy and guiding future therapeutic strategies.

## Highlights

• Transcriptomic subtyping was applied to RNAseq data from advanced EOC patients using established algorithms.
• FFPE tumor tissues yielded high-quality RNAseq data, demonstrating feasibility for transcriptomic analyses.
• Subtype classification showed significant agreement across algorithms but lacked OS differences in this patient cohort.
• Distinct immunoreactive clusters were identified, suggesting utility in stratifying patients for immunotherapy trials.
• DEGs between long- and short-term survivors highlight prognostic markers and potential therapeutic targets inEOC.

## Background

Ovarian cancer is the eighth most common type of solid cancer in women, and of those, epithelial ovarian cancer (EOC) constitutes >90 % [1]. The overall prognosis is poor due to its late symptom onset and frequent recurrence despite debulking surgery and platinum-based chemotherapy [2]. Five-year overall survival (OS) rate ranges from 92 % for local disease to 32 % for primary advanced stages, the latter comprising >80 % of the patients [3]. Approval of poly(ADP-ribose) polymerase (PARP) inhibitors has dramatically changed the course of advanced EOC, especially for patients with tumors that have pathogenic *BRCA1* or *BRCA*2 variants, and/or with defects in the homologous recombination repair-pathway, also known as HRD positive (HRDpos) [[4], [5], [6]]. These DNA based characteristics have contributed immensely to classification and treatment strategies of EOC [7]. At the RNA level, several classifiers are used in breast cancer. For example, the PAM50 classifier is routinely applied to distinguish transcriptional subtypes with prognostic and therapeutic implications [8,9]. Although on a smaller scale, several studies have attempted to identify transcriptomic subtypes of EOC using microarray-based gene expression data employing various mathematical models for algorithm design [[10], [11], [12], [13]]. Three of these classifiers were evaluated on independent datasets, revealing significant variability in subtype assignment. This issue was subsequently addressed through the development of a consensus classifier [14]. The consensus classifier is a random forest model trained on a small set of genes (*n* = 100) derived from samples consistently classified by the three previously mentioned algorithms. The subtypes identified by the consensus classifier were reported to correlate with OS, revealing an immunoreactive subtype associated with prolonged survival and a proliferative subtype linked to poor prognosis [14]. However, subtype classification has not yet become standard practice in EOC prognostication. Additionally, since these algorithms were developed on microarray data, their transferability to RNA-sequencing (RNAseq) data is a potential challenge that must be addressed.

The current analysis is based on tumor samples obtained from participants in the ENGOT-ov24/NSGO-AVANOVA1&2 (henceforth AVANOVA1&2) clinical trial, which evaluated niraparib, bevacizumab, or their combination in recurrent EOC. In short, AVANOVA2, the phase 2 part of the study, met its’ primary endpoint demonstrating a significant improvement in progression-free survival (PFS) for the combination of niraparib plus bevacizumab compared with niraparib alone, establishing this regimen as an active option in platinum-sensitive recurrent EOC [15]. The study was not powered to detect OS differences, although final survival analysis trended towards OS improvement with combination treatment [16].

With the aim of delineating EOC biology at high resolution using RNAseq, we conducted an exploratory study to investigate differences in gene expression patterns in the AVANOVA1&2 cohort between participants who become long-term survivors, and those who succumb rapidly. A key objective was to validate the feasibility of RNAseq from formalin-fixed paraffin-embedded (FFPE) EOC tissue, the most common tissue preservation method used in clinical practice. Additionally, we aimed to validate established classifiers for EOC prognosis and survival using state-of-the-art RNAseq data derived from a clinical trial cohort.

## Methods

### Patients and study design

Women with relapsed high-grade serous or endometroid ovarian cancer who were randomized in the ENGOT-ov24/NSGO-AVANOVA1&2 study (NCT02354131) [17,15] were eligible for RNAseq analysis using FFPE tumor samples preserved at the time of diagnosis. The AVANOVA1&2 study was a combined phase I and II clinical trial evaluating the effects of treatment with either niraparib, bevacizumab, or a combination of niraparib and bevacizumab at the time of relapse. This transcriptomic substudy is part of a larger translational research initiative based on AVANOVA1 and AVANOVA2, which also encompassed DNA sequencing analyses aimed at understanding PARP inhibitor response [18]. The current cohort includes the entire AVANOVA2 population, encompassing all treatment arms, including the bevacizumab monotherapy arm. This transcriptomic analysis was conducted as a *post hoc* exploratory study of data from AVANOVA1&2 conducted in accordance with the Declaration of Helsinki. An amendment describing additional sequencing analysis has been approved by institutional review boards and ethics committees of all investigational sites. Terminally ill or deceased patients were waived for participation in the study, and all other participants provided a new informed consent to participate in translational research.

### RNA sequencing

RNA was extracted from FFPE tissue, with anticipated > 50 % tumor content, and further purified using the FFPE FormaPure Protocol for RNA Isolation (Beckman Coulter, Inc., Brea, USA). In short, three tissue slices of 10µ m were deparaffinized before tissue digestion with lysis and protein kinase K. DNase I (ThermoFisher Scientific, Waltham, USA) was added to degrade DNA, and RNA was immobilized by paramagnetic beads while contaminants, including fragmented DNA, were washed away. Library preparation followed the NEBNext® Ultra™ II Directional RNA Library Prep Kit for Illumina® protocol, where 10–100 ng total RNA was diluted in nuclease-free water after determination of RNA Integrity Number (RIN) by Agilent Bioanalyzer RNA 6000 (Agilent Technologies, Santa Clara, USA). Concentration was measured using Qubit (ThermoFisher Scientific, Waltham, USA). Probes were hybridized to RNA, and rRNA was depleted before random priming. First and second strand synthesis was performed to create cDNA, which was further purified using NEBNext Sample Purification Beads. End prep was performed before adaptor-ligation, PCR amplification, and second quality check. Each RNA library was diluted into 2 nM, pooled and loaded on NovaSeq 6000 (Illumina Inc., San Diego, USA) following the NovaSeq 6000 Sequencing System Guide. Libraries were sequenced with NovaSeq 6000 SP Reagent Kit v1.5 in 300 cycles, and FASTQ-files were generated by bcl2fastq.

### Data processing

Sequencing data was generated at Department of Genomic Medicine, Rigshospitalet, Denmark. FASTQ files were aligned and quantified at gene level using star version 2.5.2b After manual quality assessment of data, the most extreme outlier on principal component 1 (PC1) and principal component 2 (PC2), respectively, were removed from further analysis.

### Gene count processing, and expression analysis

For unsupervised analysis of gene expression, final gene counts were normalized to cpm and log-transformed using the edgeR-package in R version 4.4.1 after filtering out low-count genes as described by Love et al. [19]. Heatmaps were generated based on genes with highest variance across samples and plotted using the pheatmap package. DESeq2 version 1.44.0 was used to identify DEGs in long- vs. short-term survivors [19]. Long- and short-term survivors were defined as the upper and lower interquartile range (IQR), respectively, of overall survival since diagnosis. Log fold changes were shrunken using the “ashr” method [20]. Significance level was considered with a p-value < 0.05 after adjusting for multiple testing using the Benjamini-Hochberg method. Transcriptomic subtyping algorithms were assigned on unfiltered, filtered [19], and “array-like” gene count datasets. A proxy to convert NGS data to resemble microarray data – array-like data – was applied using the voom function from the limma package [21]. The subtypes were applied using the consensusOV package in R [15]. Kaplan-Meier curves estimating OS were compared using the log rank-test with any p-value < 0.05 considered significant [22]. R (R Foundation for Statistical Computing, Vienna, Austria) version 4.3.0 was used for statistical analysis and visualization. Hierarchical clustering was performed via pheatmap (clustering_method = "ward.D", clustering_distance_cols = "euclidean", clustering_distance_rows = "manhattan") and optimal leaf ordering, using the following R function: library(seriation); function(a,b){reorder(a, dist(b, method = "manhattan"))}

## Results

### Cohort

**Fig. 1:**
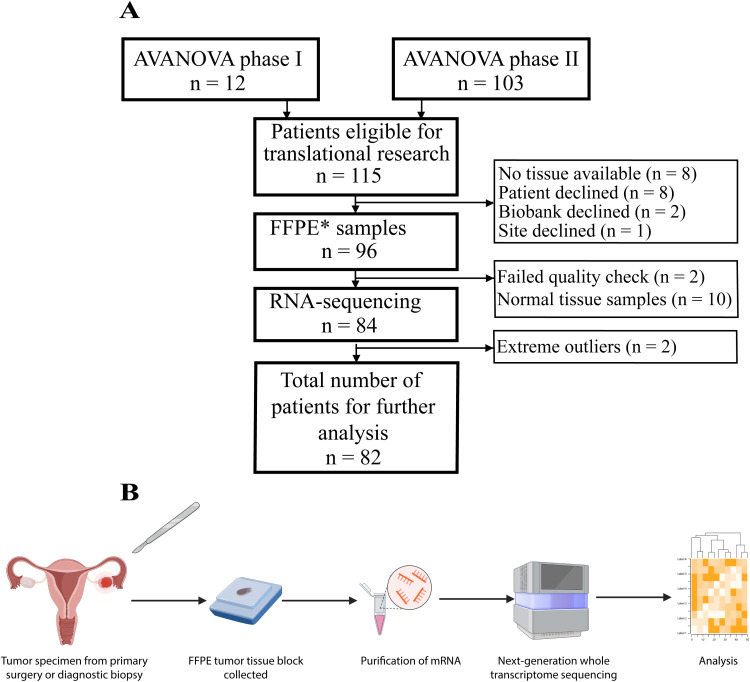
A: Consort diagram of included patients and tumor tissue samples for transcriptomic sequencing analysis. B: Tumor tissue flow from patient to analysis FFPE*: formalin-fixed paraffin-embedded.

FFPE samples were collected from 96 out of 115 eligible study participants, and 86 of these contained tumor tissue (Fig. 1A) [18]. After RNA purification 84 samples passed first quality check and were submitted to downstream analytic flow (Fig. 1B). Two outliers were removed following bioinformatic evaluation leaving RNAseq data from in total 82 individual tumor tissue samples. Patient characteristics from the time of tumor tissue preservation are shown in *Supplementary Table S1*.

### Unsupervised data visualization

**Fig. 2:**
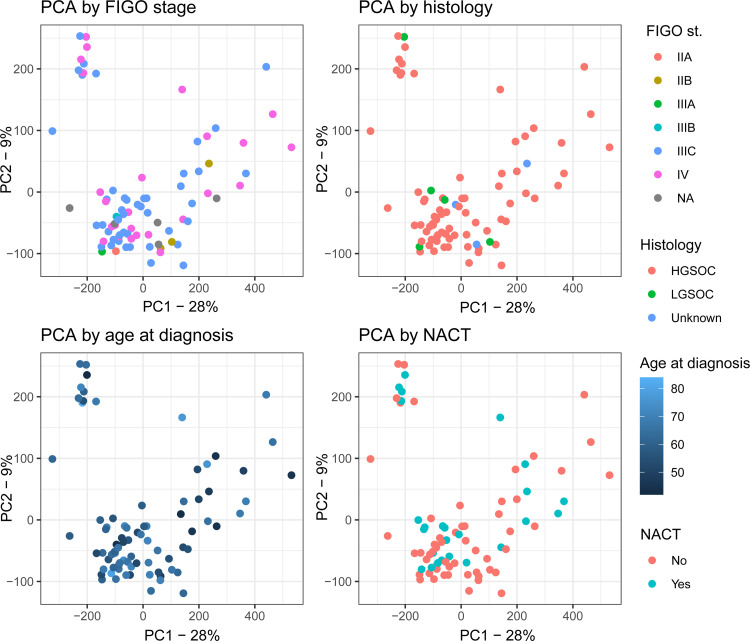
PCA plot explaining 37 % variance across samples. Samples colored by FIGO stage, histology (HGSOC = high-grade serous OC, LGSOC = low-grade serous OC), age at diagnosis, and NACT = neoadjuvant chemotherapy (y/n).

Fig. 2 illustrates an unsupervised principal component analysis (PCA) of gene expression in the samples with PC1 and PC2 together accounting for 37 % of the total variance. Separation in PC1/PC2 space is independent of known metadata such as FIGO stage, histological subtype, age, and neoadjuvant chemotherapy (NACT) (y/n), Fig. 2. Likewise, for HRD status, *BRCA1/2* mutation status, participating hospital, and overall survival length, *Supplementary Figure S1 A-D.* Similarly, subtype classifications assigned later in the analysis did not produce distinct clusters in PC1/PC2 space, *Supplementary Figure S1 E-G*.

### Unsupervised analysis of variably expressed genes

**Fig. 3:**
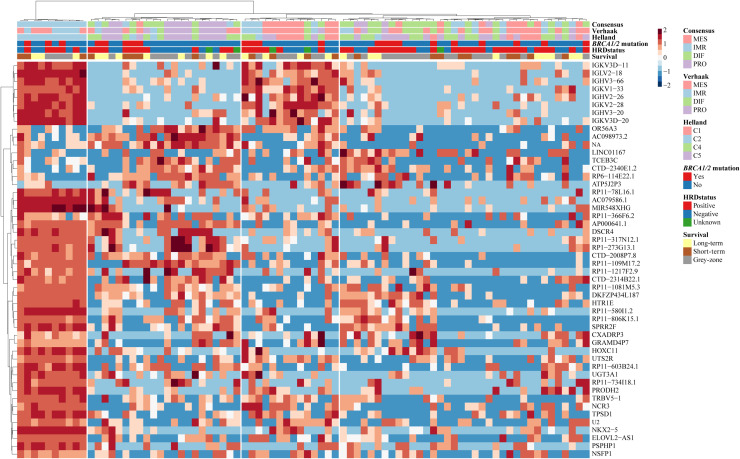
Heatmap of top 50 most variable genes. Cut-tree set to 4.

Most variably expressed genes across samples were selected to explore potential transcriptomic subtypes using hierarchical clustering. Fig. 3 displays a heatmap of the top 50 genes with highest variance across samples. *Supplementary Figure S2* and *S3* shows the top ∼1 % (*n* = 270) and top ∼10 % (*n* = 2700) variable genes, respectively. The first cluster appeared visually distinct, showing high expression of immunoglobulin family members and several long non-coding RNA fragments. Interestingly, this cluster contains almost entirely samples that were subsequently classified as immunoreactive by the subtype classifiers. Immunoglobulins are similarly upregulated in the third cluster, which is largely composed of the mesenchymal subtype. The two remaining clusters are annotated by a mix of assigned subtypes. The fourth cluster contains the highest proportion of HRDpos samples.

### DEGs in long- vs. short-term survivors

**Fig. 4:**
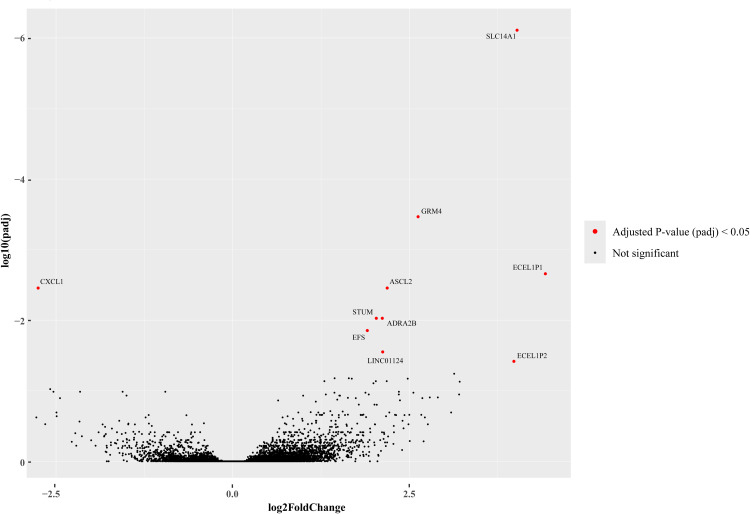
Volcano plot of DEGs.

Short-term survivors were defined as those who died within 46.5 months (∼3.9 years, 1st IQR) from time of diagnosis, while long-term survivors lived for >79 months (∼6.6 years, 3rd IQR). After adjusting for *age of diagnosis*, we identified 18 significant DEGs between long- and short-term survivors. Among these, 15 genes were significantly upregulated, and one gene downregulated, with a shrunken log2FC > 2 or < −2 (Fig. 4). A full list of DEGs is available in *Supplementary Table S2*.

DPEP3, a membrane-bound dipeptidase primarily expressed in testis and certain cancers including high-grade serous ovarian cancer [23], was significantly upregulated in long-term survivors. This was accompanied by several long and small non-coding RNAs and pseudogenes. SLC14A1, a urea transporter involved in regulating water and solute homeostasis in erythrocytes and kidneys, also showed significant upregulation in long-term survivors, with the highest log2FC aside from the pseudogenes ECEL1P1 and ECEL1P2. CXCL1, involved in chemotactic activation of neutrophils, was the only significantly downregulated gene in long-term survivors, consistent with its preciously suggested role in promoting ovarian cancer cell proliferation [23]. A heatmap of the top 50 DEGs, sorted by log2FC, is provided in *Supplementary Figure S4*.

### Classifier-subtype assignment

We applied the consensusOV classifier to perform three different subtype classification attempts using three modified datasets: the unfiltered, the filtered, and the array-like. Regardless of dataset, there was significant agreement on subtype assignment across algorithms when MES=C1 (mesenchymal), IMR=C2 (immunoreactive), DIF=C4 (differentiated), and PRO=C5 (proliferative) (χ^2^-test *p* > 0.0001, *Supplementary Table S4*). Confusion plots present robustness in assignment between the Helland and Consensus algorithm for filtered vs. unfiltered data, and in the Consensus algorithm for unfiltered vs. filtered, and unfiltered vs. array-like data (*Supplementary Figure S5A-C*). Cases where all three algorithms agreed were highest when applied to the array-like dataset (*n* = 50/82) compared to the filtered and unfiltered data (*n* = 38/82 vs. 39/82, respectively). Subtype assignment with at least two agreements between algorithms were comparable for all datasets (from *n* = 74/82 to *n* = 81/82). The level of agreement measured by Cohen’s Kappa ranges from 0.33 between the Helland and Verhaak algorithms applied to unfiltered data to 0.73 when applied to array-like data indicating a *minimal to moderate level of agreement* [24]. Venn diagrams showing overlapping subtype assignment regardless of algorithm are provided in *Supplementary Figure S6*.

### Overall survival between subtypes

We analyzed survival from diagnosis on the unfiltered, filtered and array-like subtypes (*Supplementary Figure S7, S8, and S9*). Contrary to previous findings, no statistically significant differences in OS were observed between subtypes in this cohort, regardless of the classification method used. We subsequently identified samples that were uniformly classified regardless of algorithm – where MES=C1, IMR=C2, DIF=C4, and PRO=C5 – but again found no differences across these subtypes (*Supplementary Figure S10*).

## Discussion

Transcriptomic subtype classification has not yet been integrated as a standard approach in the clinical management or prognostication of EOC. Nevertheless, there remains a significant need to enhance treatment strategies and refine prognostic tools. In this study, we successfully utilized FFPE tumor tissue for total RNAseq, achieving high sample quality with the majority of specimens passing quality control.

Unsupervised PCA revealed distinct variance in gene expression; however, this variance did not fully correlate with clinical metadata, nor with subtype classifications assigned using existing algorithms. Most variable genes across samples displayed a distinct cluster characterized by the upregulation of immunoglobulins, assigned to an immunoreactive subtype by all three established classifiers. Three other unsupervised clusters were identified but did not reveal the same distinct differentiation as the immunogenic cluster.

Until now, immune checkpoint inhibitor treatment in ovarian cancer has been discouraging with negative PFS and OS results in clinical trials despite analyzing participants with PD-L1 positive tumors separately [[25], [26], [27]]. Prospectively stratifying for an immunoreactive tumor type by their transcriptomic profile in clinical trials evaluating immunotherapy has the potential to define a more responsive OC subgroup.

Subtype classification of EOC tumors is still undergoing refinement. In this study, we used the consensusOV package in R to apply transcriptomic subtype algorithms—originally developed for microarray data—to RNAseq data. We tested three approaches: using raw gene counts, filtering out low-count genes, and transforming RNAseq data to mimic microarray profiles. Subtype classification was feasible with all three methods. While we observed statistically significant concordance in subtype assignment across these dataset formats, notable inconsistencies also occurred. Similarly, subtype classifications often differed when using different algorithms. These findings highlight the challenge of achieving consistent subtype assignments across both different algorithms and various RNAseq data processing methods.

Contrary to expectations, this study did not find significant differences in OS between the subtypes. Recent work has addressed the same challenge applying subtype classifiers based on array-data to RNAseq data finding robustness in subtype assignment, indicating that the delineation of subtypes is agnostic for sequencing technology [28]. However, consistent with our findings, that study also did not show clear survival differences between subtypes. Additionally, revisiting datasets used for original subtype development and excluding samples of borderline type resulted in the identification of two or three subtypes rather than four, which have been the consensus between early subtype classification models [29]. When assigning three subtypes, the differentiated and the immunoreactive subtypes collapsed into the same cluster, whereas the mesenchymal and the proliferative were still distinct. In our study, the immunoreactive subtype was the most distinct.

Significant DEGs between long- and short-term survivors included upregulation of DPEP3. Targeting DPEP3 with and antibody-drug conjugate (ADC), SC-003, has previously been explored in a phase 1a/1b study demonstrating ORR of 4 % and no durable responses [30]. Together with a high toxicity profile, further development of SC-003 was halted. Likewise, SLC14A1 was highly expressed in long-term survivors in this EOC cohort. A study investigating SLC14A1 in prostate cancer showed a correlation between high expression and increased time for biochemical relapse [31], suggesting the gene to be associated with better prognosis across different solid tumors. ASCL2 was similarly upregulated in long-term responders in our cohort in contrast to the identification of the gene expression to be a poor prognostic marker in breast cancer patients [32]. Upregulation of CXCL1 has increased proliferation of ovarian cancer cells in a cell line study, while downregulation was found to be inhibitory, which supports our observation of significantly lower CXCL1 expression in long-term survivors [33]. None of the significant DEGs identified in this analysis act in the homologous recombination pathway, which—when impaired—is known to be associated with both PARPi benefit and long-term prognosis. Likewise, the unsupervised analyses did not show distinct clustering by HRD status. Several explanations for this lack of findings can be proposed, including tissue heterogeneity that may hide molecular signals present only in the tumor cells. It also suggests that the transcriptomic profile of HRD tumors is heterogeneous in itself. Despite this, the identification of survival-associated up- and downregulated genes may inform future ADC development and highlights the value of RNAseq in biomarker discovery. Other research groups have explored the potential of single-cell and spatial transcriptomics to identify prognostic subtypes and DEGs [[34], [35], [36]].

Although PFS is a clinically relevant endpoint in ovarian cancer and AVANOVA2 demonstrated a marked treatment effect on PFS, we did not perform a separate DEG analysis based on PFS in this exploratory study. Given the strong arm-dependent PFS differences, any transcriptomic associations would be heavily confounded by treatment exposure, and a robust PFS-based biomarker analysis would require a larger cohort with stratified or treatment-adjusted models. Such an analysis would constitute a separate translational project and lies beyond the scope of the current OS-focused manuscript.

In conclusion, in this study we identify significantly deregulated genes likely involved in mechanisms influencing prognosis and potential drug targets. Additionally, we highlight the complexity and poor generalizability of transcriptomic subtyping for EOC across sequencing platforms. Although data were derived from a clinical trial cohort, ensuring robustness, this study has several important limitations that should be acknowledged when interpreting the findings. First, the overall cohort size (*n* = 82) restricts statistical power, particularly for detecting significant survival differences between the four transcriptomic subtypes. While we attempted to validate subtype-survival associations described in prior work, the sample size is likely insufficient to capture more subtle prognostic differences. Second, the cohort represents a heterogeneous population with respect to treatment exposure. Participants in AVANOVA1&2 received niraparib, bevacizumab, or the combination, and these treatment modalities are known to influence both PFS and OS. Furthermore, the post hoc design of this translational substudy prevent adjusting for treatment exposure outside the AVANOVA1&2 trial, and the potential for confounding by therapeutic heterogeneity is substantial and should be considered when interpreting the absence of survival differences. Third, despite uniform processing pipelines, intra-tumoral heterogeneity and the application of microarray-derived classifiers to RNAseq data likely contribute with technical challenges. Together, these limitations underscore that our observations should be interpreted as hypothesis-generating and warrant validation in larger, more uniform, prospectively collected patient cohorts. Future studies may aim to refine transcriptomic classifiers and explore the molecular mechanism behind subtype-specific survival, to enhance prognostic accuracy, devise a generally applicable subtyping scheme, and guide therapeutic decisions in EOC.

## Data availability

The RNAseq data generated in this study are not publicly available due to patient privacy requirements.

## Declaration of generative AI and AI-assisted technologies in the writing process

During the preparation of this work the authors used ChatGPT 4o in order to improve readability, clarity, and language precision. After using this tool, the authors reviewed and edited the content as needed and take full responsibility for the content of the published article.

## CRediT authorship contribution statement

**Maj K Kjeldsen:** Writing – original draft, Visualization, Validation, Project administration, Methodology, Investigation, Funding acquisition, Formal analysis, Data curation, Conceptualization. **Frederik Otzen Bagger:** Writing – review & editing, Writing – original draft, Visualization, Validation, Supervision, Methodology, Formal analysis, Conceptualization. **Henrik Roed:** Writing – review & editing, Investigation, Data curation. **Gitte-Bettina Nyvang:** Writing – review & editing, Investigation, Data curation. **Charlotte Aaquist Haslund:** Writing – review & editing, Investigation, Data curation. **Anja Oer Knudsen:** Writing – review & editing, Investigation, Data curation. **Anne Krejbjerg Motavaf:** Writing – review & editing, Investigation, Data curation. **Susanne Malander:** Writing – review & editing, Investigation, Data curation. **Maarit Anttila:** Writing – review & editing, Investigation, Data curation. **Gabriel Lindahl:** Writing – review & editing, Investigation, Data curation. **Johanna Mäenpää:** Writing – review & editing, Investigation, Data curation. **Maria Dimoula:** Writing – review & editing, Investigation, Data curation. **Theresa Werner:** Writing – review & editing, Investigation, Data curation. **Trine Zeeberg Iversen:** Writing – review & editing, Investigation, Data curation. **Sakari Hietanen:** Writing – review & editing, Investigation, Data curation. **Lars Fokdal:** Writing – review & editing, Investigation, Data curation. **Hanna Dahlstrand:** Writing – review & editing, Investigation, Data curation. **Line Bjørge:** Writing – review & editing, Investigation, Data curation. **Michael Birrer:** Writing – review & editing, Investigation, Data curation. **Mansoor Raza Mirza:** Writing – review & editing, Validation, Supervision, Project administration, Methodology, Investigation, Funding acquisition, Data curation, Conceptualization. **Maria Rossing:** Writing – review & editing, Writing – original draft, Visualization, Validation, Supervision, Methodology, Formal analysis, Conceptualization.

## Declaration of competing interest

All authors except for MKK, FOB, and MR were investigators of ENGOT-ov24/NSGO-AVANOVA2 and are members of NSGO—CTU. Personal COIs listed below:

MKK: Research grant from GSK. Member of NSGO—CTU.

FOB: Honoraria and consultation fees from AstraZeneca. Real owner of FOBinf ApS via FOB Holding ApS, advisory of Immunitrack ApS (owned by Eli Lilly), Aida Oncology, Hervolusion ApS, InProther Aps

GBN: Travel and registration fee to ESGO 2024 (GSK)

CH: invited speaker (MSD, BMS and GSK), Advisory Board and invited speaker (AZ)

MA: advisory board (MSD and Eisai)

GL: invited speaker (GSK and AZ)

JM: honoraria (Eisai, GSK, AZ, GSK)

SH: consulting or advisory role (AZ, GSK, MSD, EISAI, ORION)

HD: invited speaker (GSK and Roche), writing engagement (Roche), advisory board (AZ)

LB: advisory board and research grant (AZ), invited speaker (GSK and MSD)

MM: Leadership (Karyopharm Therapeutics, Sera Prognostics), Stock and Other Ownership Interests (Karyopharm Therapeutics, Sera Prognostics), Honoraria (Roche, AstraZeneca, Genmab/Seattle Genetics, GlaxoSmithKline, Merck, Mersana, Takeda, Zai Lab, Geneos, Allarity Therapeutics), Consulting or Advisory Role (AstraZeneca, Genmab, Karyopharm Therapeutics, Pfizer, GlaxoSmithKline) Research Funding (AstraZeneca (Inst), Boehringer Ingelheim (Inst), Pfizer (Inst), Tesaro (Inst), Clovis Oncology (Inst), Ultimovacs (Inst), Apexigen (Inst), GlaxoSmithKline (Inst)), Travel, Accommodations, Expenses (AstraZeneca, Karyopharm Therapeutics, Pfizer, Roche, Tesaro, SeraCare)

MR: Consulting or Advisory Role (AstraZeneca, MSD), Research Funding (AstraZeneca (Inst))

All other authors declare no conflicts of interest.

## Appendix Supplementary materials

Image, application 1
