# Supplementary material for: Exploring survival-associated transcriptomic subtypes in ovarian cancer using RNAseq from FFPE tissues in a clinical trial cohort
Source: Transl Oncol. 2026 Mar 19;67:102740. doi: 10.1016/j.tranon.2026.102740 (PMC13018888; doi:10.1016/j.tranon.2026.102740)
Supplement: Supplementary file 1 [file mmc1.docx]

### Supplementary material

| **Characteristic** | **N = 82**^1^ |
| --- | --- |
| Age at diagnosis (y) | 63 (42-84) |
| FIGO stage |  |
| IIA | 1 |
| IIB | 3 |
| IIIA | 1 |
| IIIB | 1 |
| IIIC | 46 |
| IV | 25 |
| Unknown | 5 |
| Histological subtype |  |
| High-grade | 74 |
| Low-grade | 5 |
| Unknown | 3 |
| Received neoadjuvant platinum | 25 |
| HRD status by myChoice |  |
| Positive | 46 |
| Negative | 31 |
| Unknown | 5 |
| BRCA1/2 mutations |  |
| BRCA1m | 12 |
| BRCA2m | 10 |
| BRCAwt | 60 |

| ^1^Median (Minimum-Maximum); n |
| --- |

Supplementary Table S1: Patient characteristics


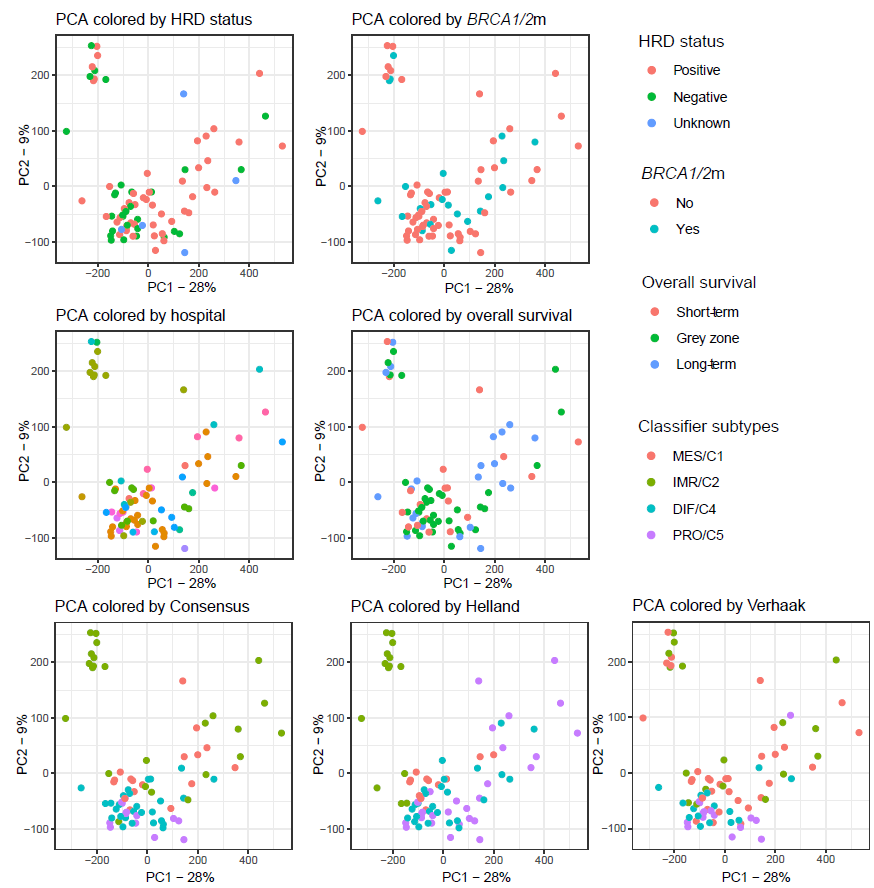


Supplementary Figure S1 A-G: PCA-plots with PC1 explaining 28% variance and PC2 explaining 9%. Samples are colored by different metadata – see legends for explanation.

###
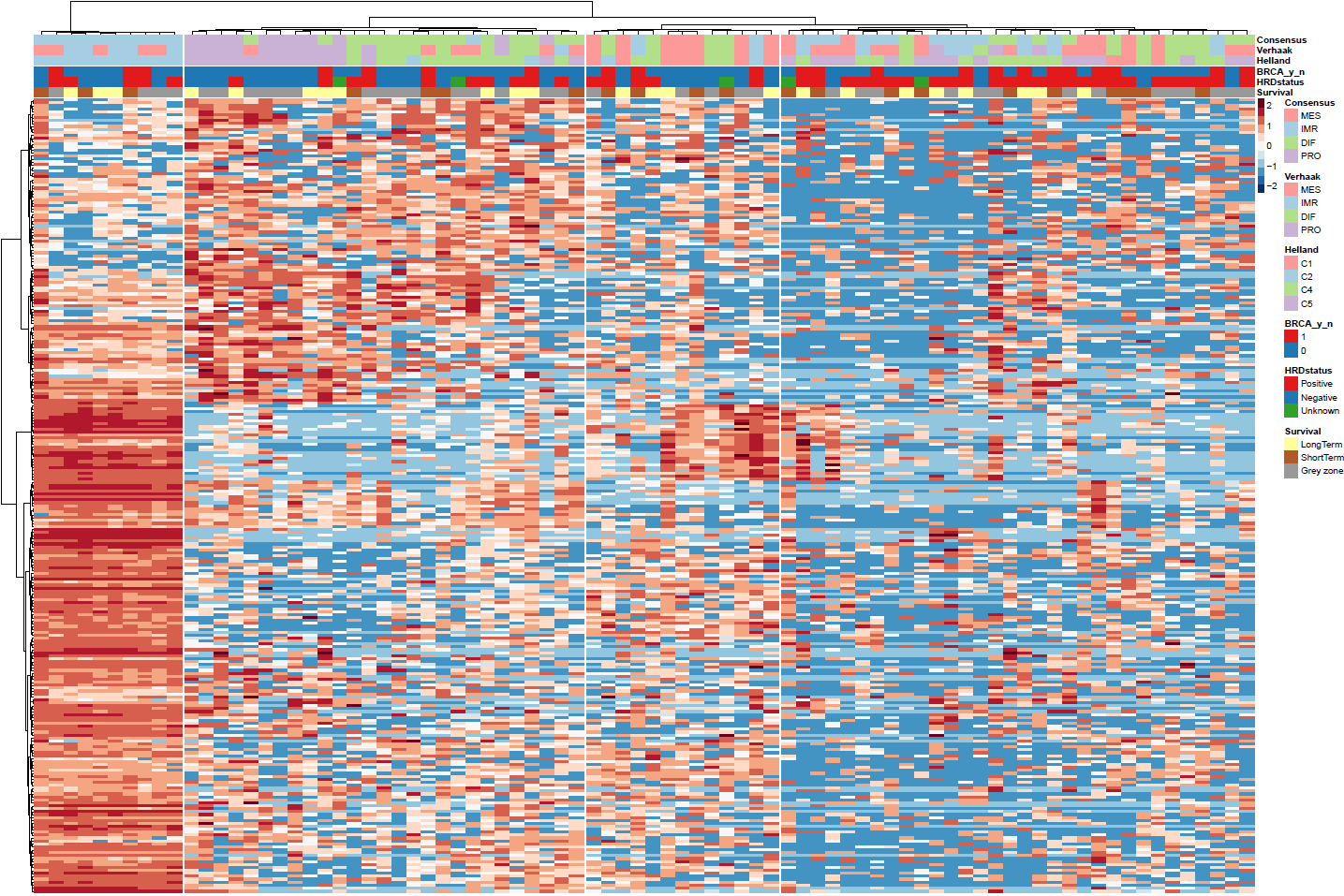


Supplementary Figure S2: Top 1% variable genes.


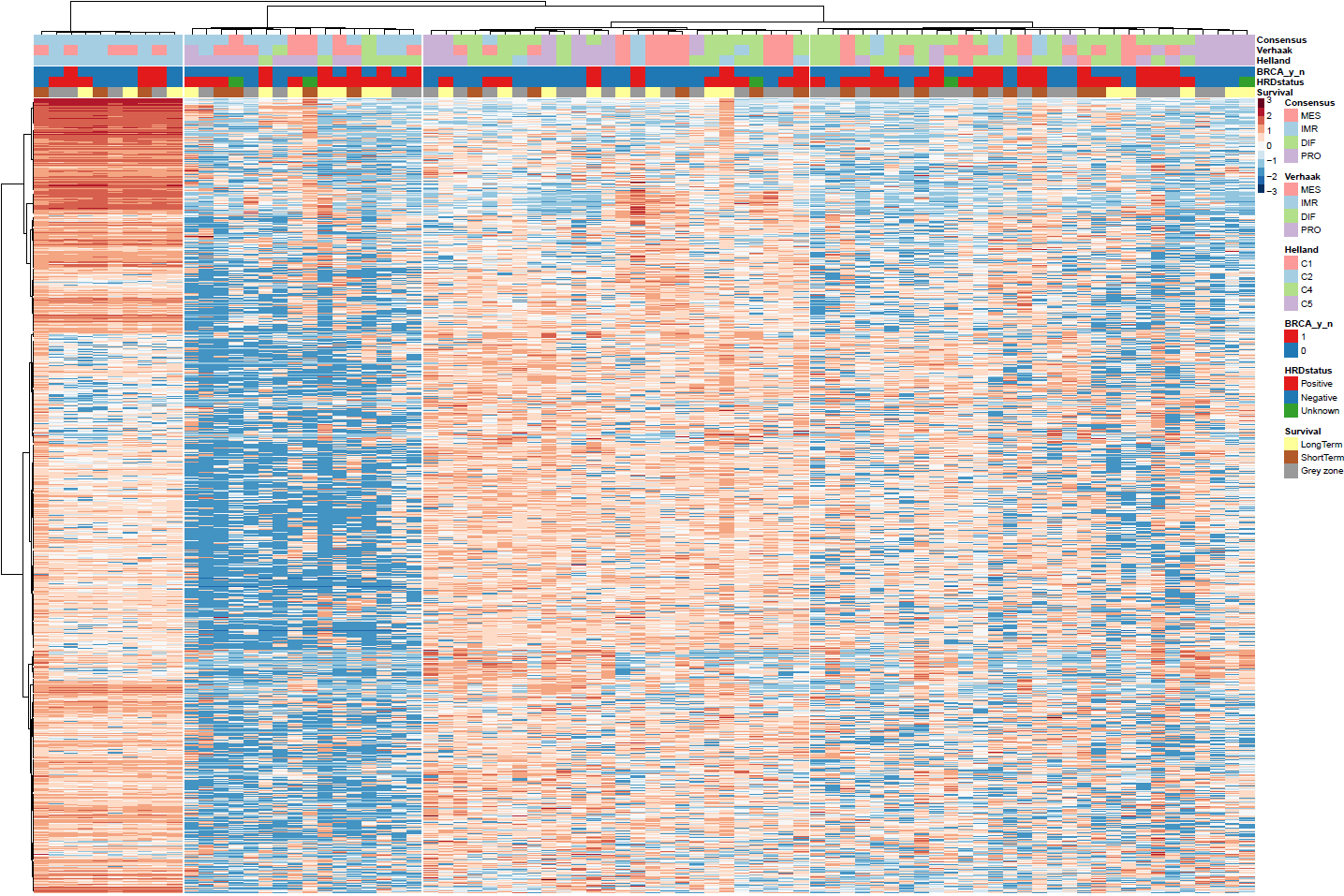


Supplementary Figure S3: Top 10% variable genes.

| symbol | baseMean | log2FoldChange | padj | full name | function |
| --- | --- | --- | --- | --- | --- |
| SLC14A1 | 109,343777 | 3,983674892 | 1,99962E-06 | Solute Carrier Family 14 (Urea Transporter) | transport of urea and water across the cell membrane of erythrocytes |
| ADRA2B | 79,04454 | 2,485032882 | 0,000127072 | Adrenoceptor Alpha 2B | mediate the catecholamine-induced inhibition of adenylate cyclase; central nervous system and sympathic nerve system |
| ECEL1P1 | 49,0361485 | 4,680165073 | 0,000295259 | Endothelin Converting Enzyme Like 1 Pseudogene 1 | pseudogene |
| GRM4 | 154,033509 | 2,591626884 | 0,000295259 | Glutamate Metabotropic Receptor 4 | inhibits adenylate cyclase activity |
| EFS | 398,130902 | 2,154305154 | 0,000585328 | Embryonal Fyn-Associated Substrate | Docking protein which plays a central coordinating role for tyrosine-kinase-based signaling related to cell adhesion |
| ASCL2 | 41,6434488 | 2,280931954 | 0,000624068 | Achaete-Scute Family BHLH Transcription Factor 2 | transcription factor |
| ECEL1P2 | 24,1214871 | 4,611990233 | 0,001383872 | Endothelin Converting Enzyme Like 1 Pseudogene 2 | pseudogene |
| KCNN1 | 36,0980972 | 2,420684982 | 0,002170736 | Potassium Calcium-Activated Channel Subfamily N Member 1 | voltage-independent potassium channel |
| CXCL1 | 69,443794 | -2,706514931 | 0,003195208 | C-X-C Motif Chemokine Ligand 1 | chemotactic activity for neutrophils |
| STUM | 193,523113 | 2,056079903 | 0,003727678 | Stum, Mechanosensory Transduction Mediator Homolog | Predicted to be integral component of membrane |
| HMGA2 | 539,38089 | 2,203192207 | 0,009357621 | High Mobility Group AT-Hook 2 | may act as a transcriptional regulating factor |
| RP11-923I11.6 | 117,038143 | 2,203504037 | 0,015022124 | NA | long non-coding RNA |
| SNORD3B-2 | 289,627839 | 2,433561375 | 0,024260798 | Small Nucleolar RNA, C/D Box 3B-2 | small non-coding nucleolar RNAs |
| ALPPL2 | 140,932399 | 3,419441684 | 0,030311327 | Alkaline Phosphatase, Germ Cell | hydrolyze various phosphate compounds |
| DPEP3 | 51,2522177 | 3,133103514 | 0,040209351 | Dipeptidase 3 | lacks dipeptidase activity; expressed in testis and high-grade serous OC |

Supplementary Table S2: Significantly over- and under-expressed genes in long- vs. short-term survivors =DEGs.


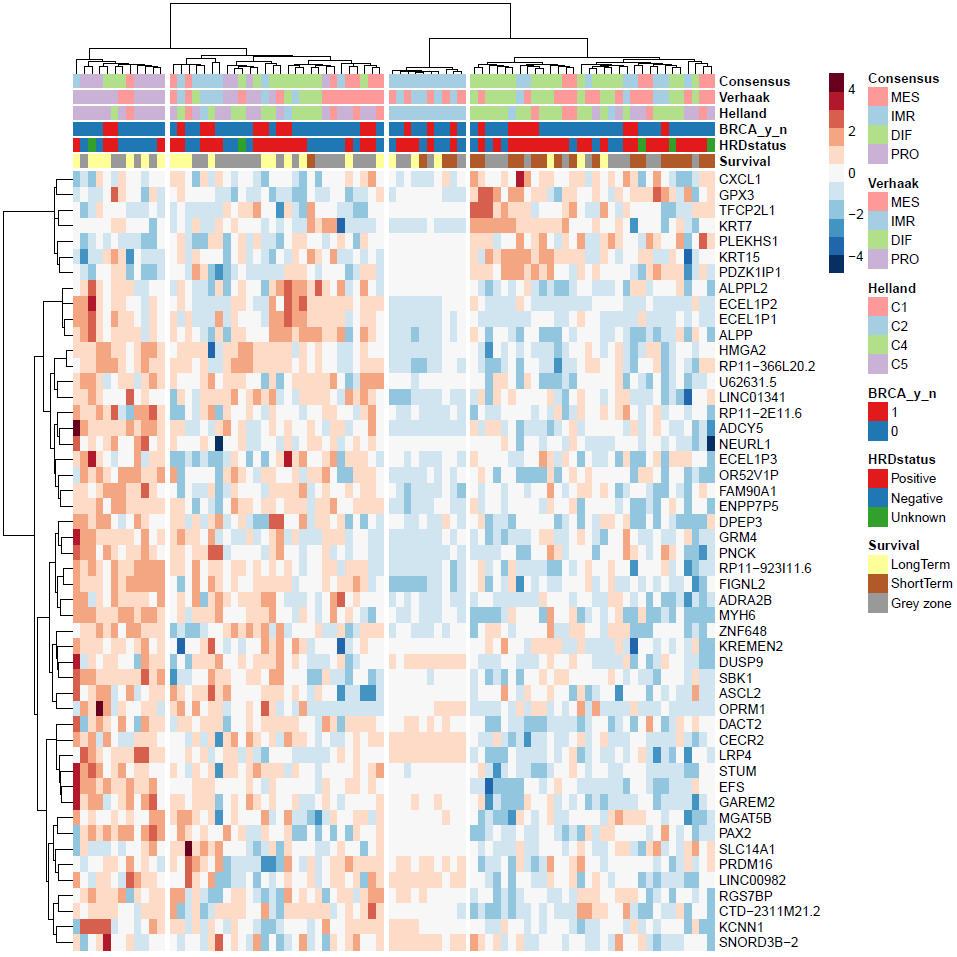


Supplementary Figure S4: Top 50 DEGs in long- vs. short-term survivors

Filtered data:

|Metric |Value |

|:----------------------------------------------|:-------|

|All Three Agree Count |38 |

|At Least Two Agree Count |75 |

|Cohen's Kappa (Consensus vs Helland) |0.5 |

|Cohen's Kappa (Consensus vs Verhaak) |0.61 |

|Cohen's Kappa (Helland vs Verhaak) |0.37 |

|Chi-Square Test p-value (Consensus vs Helland) |<0.0001 |

|Chi-Square Test p-value (Consensus vs Verhaak) |<0.0001 |

|Chi-Square Test p-value (Helland vs Verhaak) |<0.0001 |

Unfiltered data:

|Metric |Value |

|:----------------------------------------------|:-------|

|All Three Agree Count |39 |

|At Least Two Agree Count |74 |

|Cohen's Kappa (Consensus vs Helland) |0.5 |

|Cohen's Kappa (Consensus vs Verhaak) |0.66 |

|Cohen's Kappa (Helland vs Verhaak) |0.33 |

|Chi-Square Test p-value (Consensus vs Helland) |<0.0001 |

|Chi-Square Test p-value (Consensus vs Verhaak) |<0.0001 |

|Chi-Square Test p-value (Helland vs Verhaak) |<0.0001 |

Array-like data:

|Metric |Value |

|:----------------------------------------------|:-------|

|All Three Agree Count |50 |

|At Least Two Agree Count |81 |

|Cohen's Kappa (Consensus vs Helland) |0.63 |

|Cohen's Kappa (Consensus vs Verhaak) |0.58 |

|Cohen's Kappa (Helland vs Verhaak) |0.73 |

|Chi-Square Test p-value (Consensus vs Helland) |<0.0001 |

|Chi-Square Test p-value (Consensus vs Verhaak) |<0.0001 |

|Chi-Square Test p-value (Helland vs Verhaak) |<0.0001 |

*Supplementary Table S4*: Agreements in tables with Cohen’s Kappa between algorithms and p-value for statistically significant agreement on subtypes.

A:
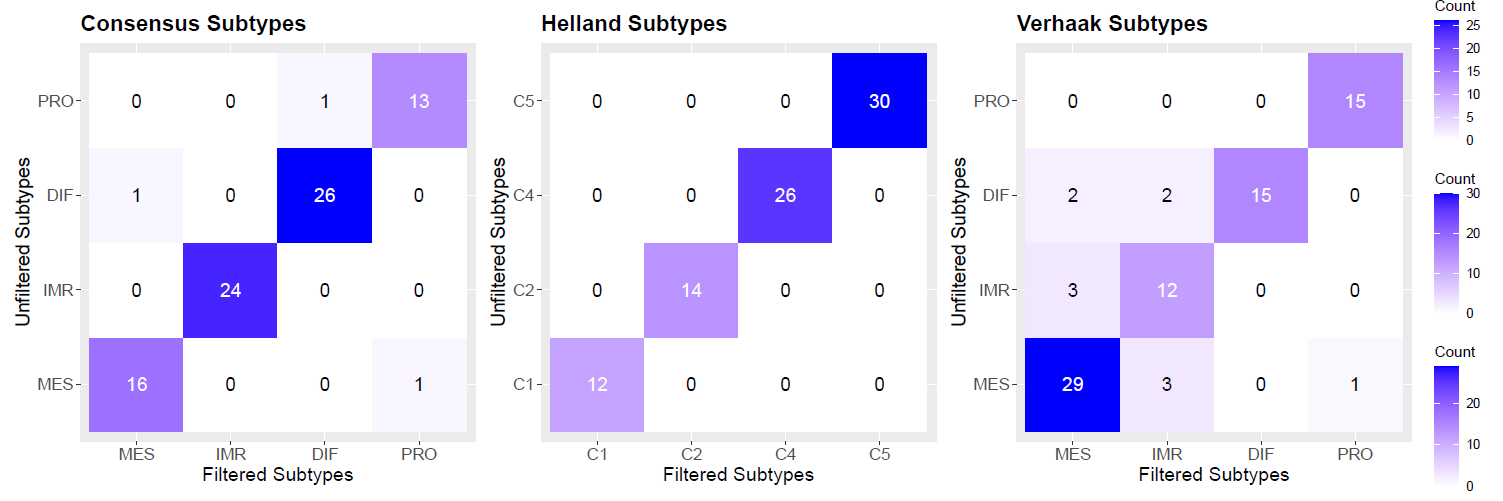


B:
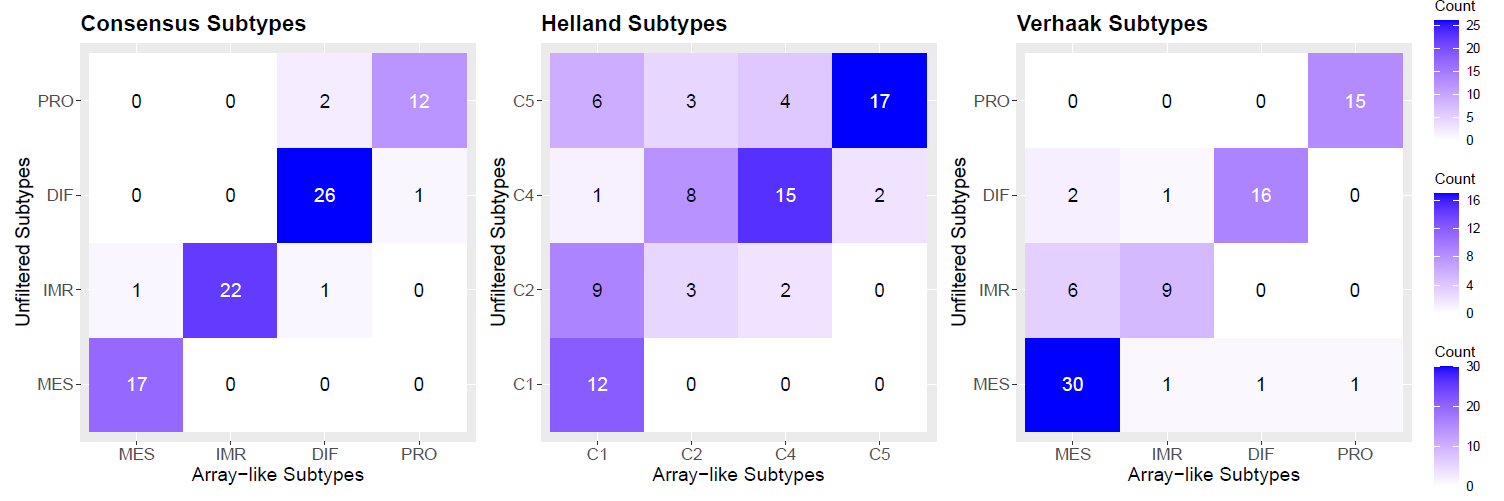


C:
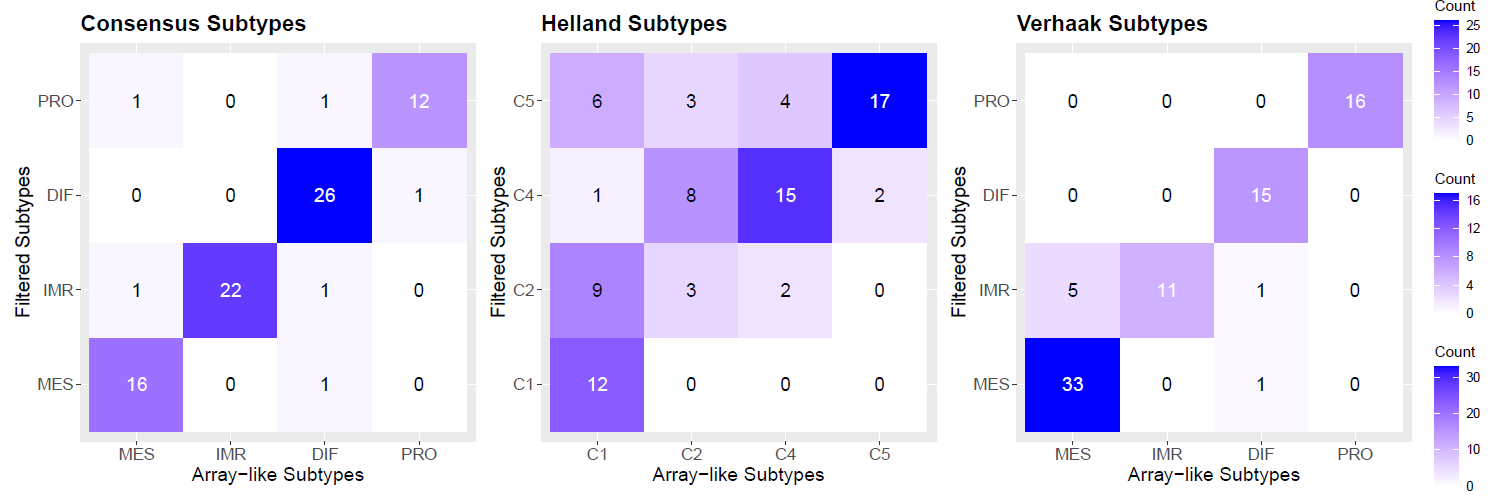


Supplementary Figure S5 A-C: Confusion plots of agreements within each algorithm for filtered, unfiltered, and “array-like” gene count data.


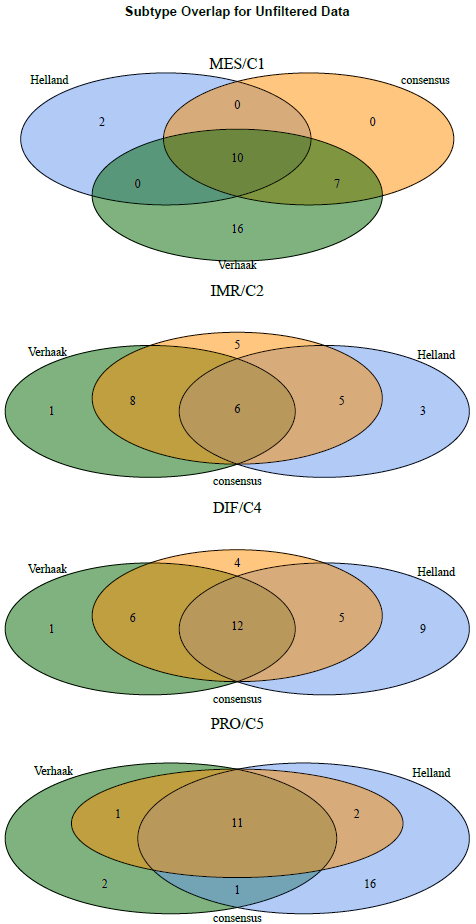

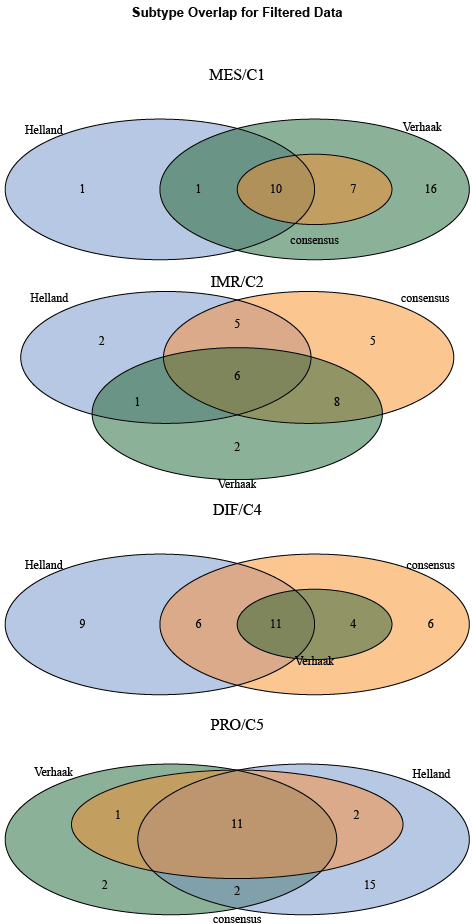

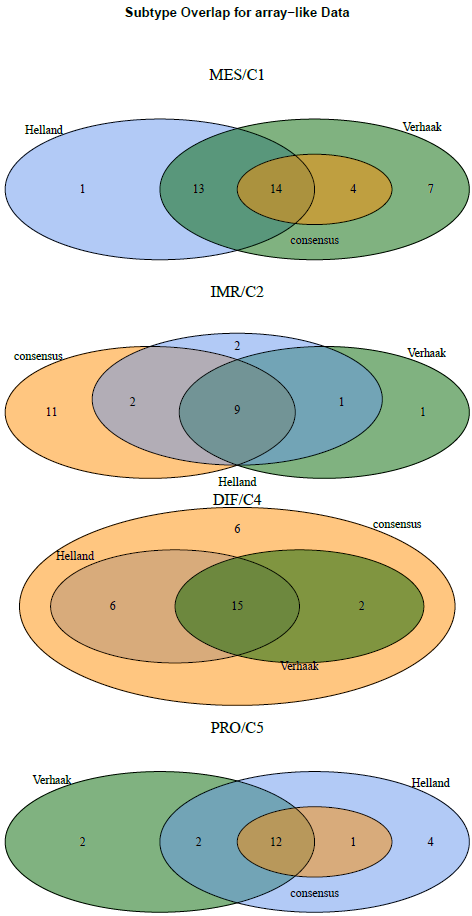


Supplementary Figure S6: Venn diagrams of agreements between algorithm for each subtype both for unfiltered date (left), filtered data (middle), and array-like data (right).


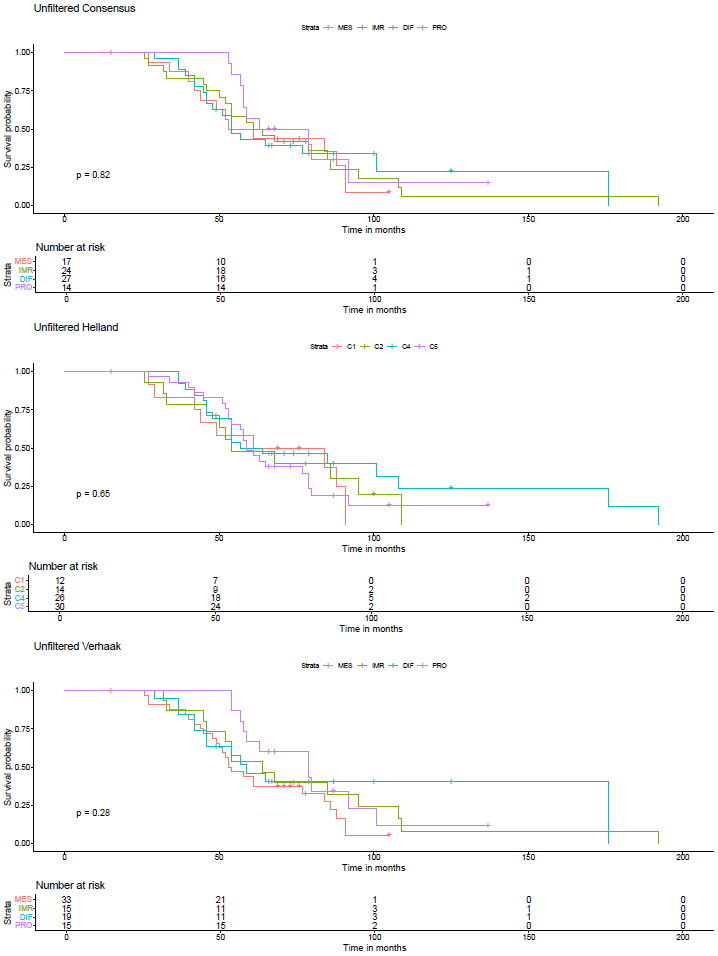


Supplementary figure S7: Transcriptomic subtypes assigned using the unfiltered RNAseq dataset with regards to OS.


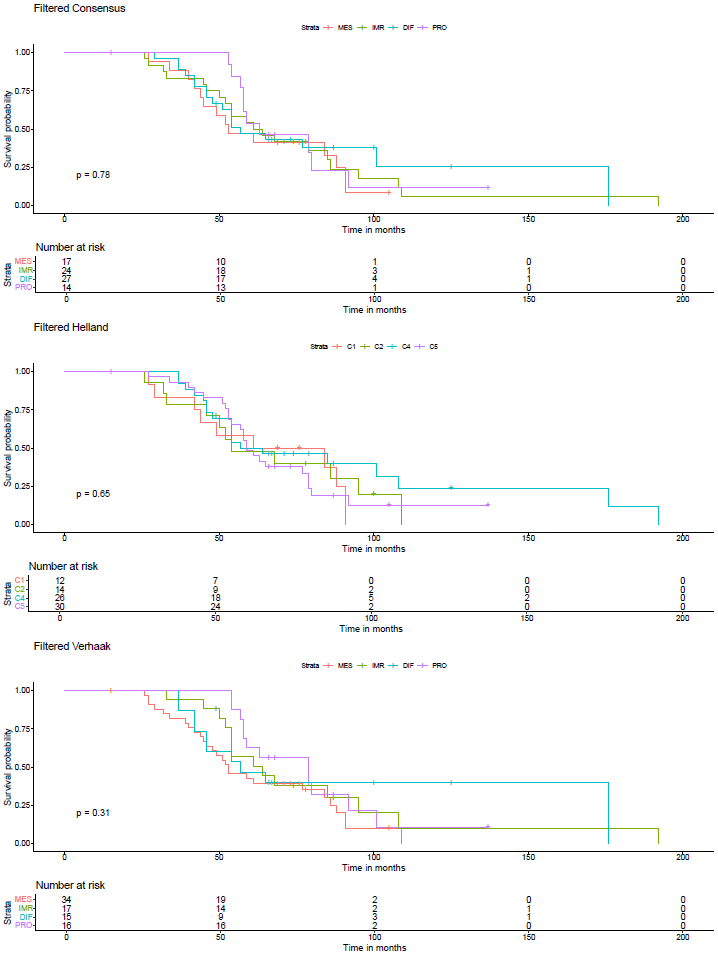


Supplementary figure S8: Transcriptomic subtypes assigned using the filtered RNAseq dataset with regards to OS.


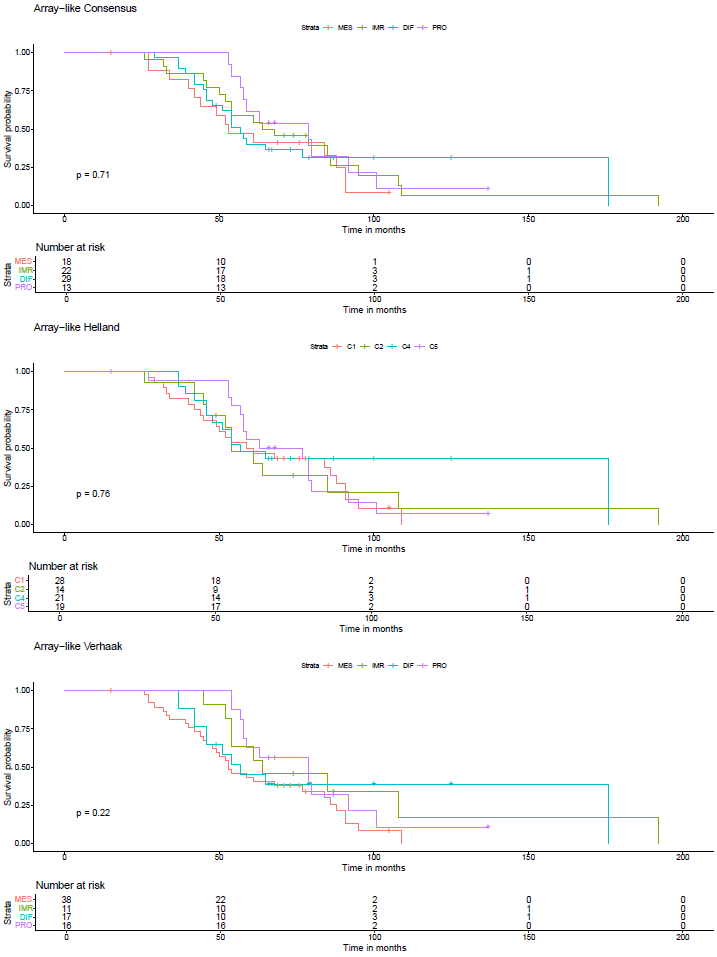


Supplementary figure S9: Transcriptomic subtypes assigned using the array-like RNAseq dataset with regards to OS.


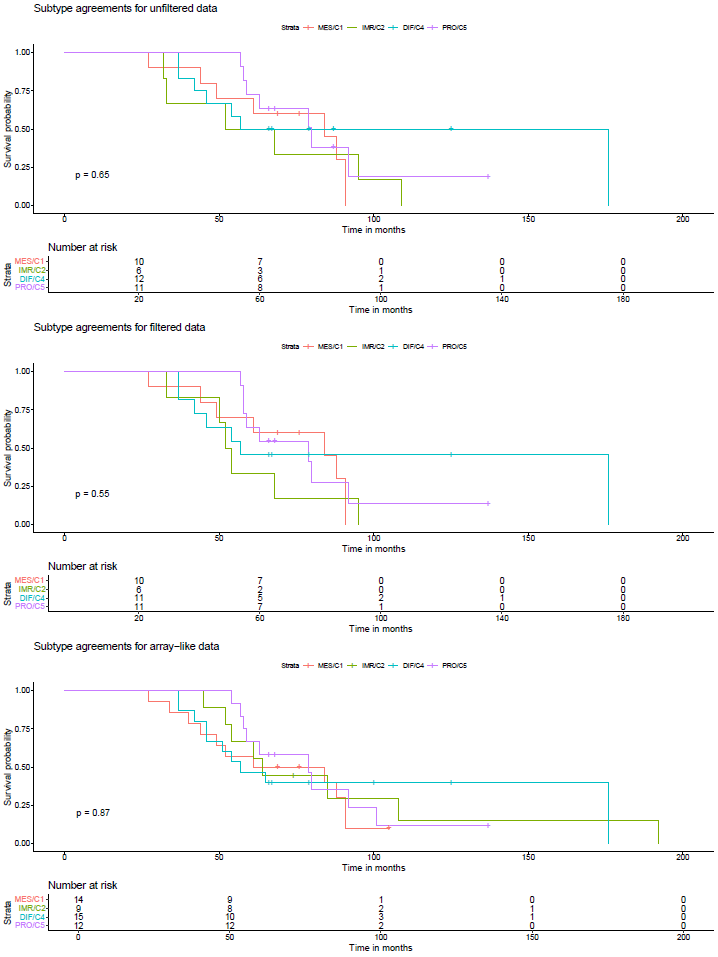


Supplementary figure S10: Kaplan-Meier curves for OS based on subtype agreement between the algorithms within each dataset.
